# *Candida* Bezoars with Urinary Tract Obstruction in Two Women without Immunocompromising Conditions

**DOI:** 10.1100/tsw.2011.119

**Published:** 2011-06-09

**Authors:** Giuseppe Di Paola, Andrea Mogorovich, Girolamo Fiorini, Maria Giuseppa Cuttano, Francesca Manassero, Cesare Selli

**Affiliations:** Department of Urology, University of Pisa, Pisa, Italy

**Keywords:** Candida bezoars, mycetoma, urinary tract obstruction, immunosuppression

## Abstract

More than half of the cases of fungal infections of the urinary tract are caused by *Candida* sp., but occurrence of obstructive uropathy caused by mycetomas or fungus balls (urobezoars) is extremely rare. The latter are conglomerates of fungal hyphae. Diabetes mellitus, immunosuppression, chronic disease, and malignancies are known predisposing factors. Preoperative imaging is not pathognomonic; blood clots, radiolucent urinary calculi, air bubbles, and inflammatory debris can mimic urobezoars. We report on two otherwise healthy women presenting with urinary tract obstruction caused by candidal mycetomas of the renal pelvis that mimicked matrix lithiasis.

## INTRODUCTION

Fungal infections involving the urinary tract are not rare[1]; the most common cause is *Candida albicans*, which is responsible for more than half of the cases[2]. *C. albicans* is a commensal organism found frequently on the skin and gastrointestinal, respiratory, and lower genitourinary tracts[3]. In an immunocompromised or debilitated host, this fungus may become a pathogen; diabetes mellitus and various diseases leading to immunosuppressive or debilitating conditions constitute predisposing factors. In such cases, two different modalities of infection may occur: the systemic candidiasis results from disseminated infection with involvement of multiple organs, possibly leading to death usually due to renal failure; less severe is the occurrence of the primary renal candidiasis with infection limited to the kidney[4]. Urinary tract colonization can be due either to hematogenous dissemination of the organism or a retrograde infection. The fungi extend into the collecting system and rarely coalesce to form bezoars or fungus balls, which can cause hydronephrosis and obstructive uropathy[5]. Radiologic features of fungus balls, although characteristics, are not pathognomonic and can be mimicked by blood clots, radiolucent urinary calculi, air bubbles, inflammatory debris, and transitional cell carcinoma; the typical aspect is an intraluminal filling defect of the drainage system sometimes leading to obstruction. On ultrasound, the fungus balls are usually hyperechoic lesions of the collecting system, but less frequently they can result as hypoechoic. In debilitated or immunosuppressed patients, when upper urinary tract obstruction with this radiologic pattern occurs, fungal infection should be considered and a urine specimen culture is advisable. When a fungal urinary tract infection is diagnosed, a number of therapeutic options for the treatment of renal mycetoma exists. Parenteral antifungal drug treatment is the first option, even if additional local irrigation of the fungus balls with antifungal drugs through a percutaneous catheter has been shown to be an effective way of treatment[6]. However, when facing cases of a mycetoma mimicking other diseases amenable to surgical treatment or drug-resistant infections, surgical removal of the fungus ball must be considered. We report on two women, without conventional risk factors, presenting with urinary tract obstruction caused by candidal mycetomas of the renal pelvis that mimicked matrix lithiasis.

## CASE 1

A 74-year-old woman, previously submitted to open, left-sided pyelolithotomy and multiple ESWL (extracorporeal shock wave lithotripsy) treatments for right-kidney urolithiasis, was referred to our department for right flank pain and fever. Ultrasound imaging showed the presence of hyperechoic lesions in the right renal pelvis with posterior acoustic shadowing; on CT urography, the pelvis contained gas bubbles, an extensive low-density filling defect of the collecting system, and calcified areas (Fig. 1). The discrepancy between the filling defect and the size of the calcifications led us to hypothesize the presence of matrix lithiasis. Right-sided, open pyelotomy was performed with complete removal of abundant, brown colored, and amorphous material (Fig. 2); definitive histology showed a mycetoma caused by *C. albicans*. The patient was administered antifungal treatment with fluconazole 400 mg daily for 1 month. After 12 months, the patient was asymptomatic, without recurrent fungal infection on urine culture.

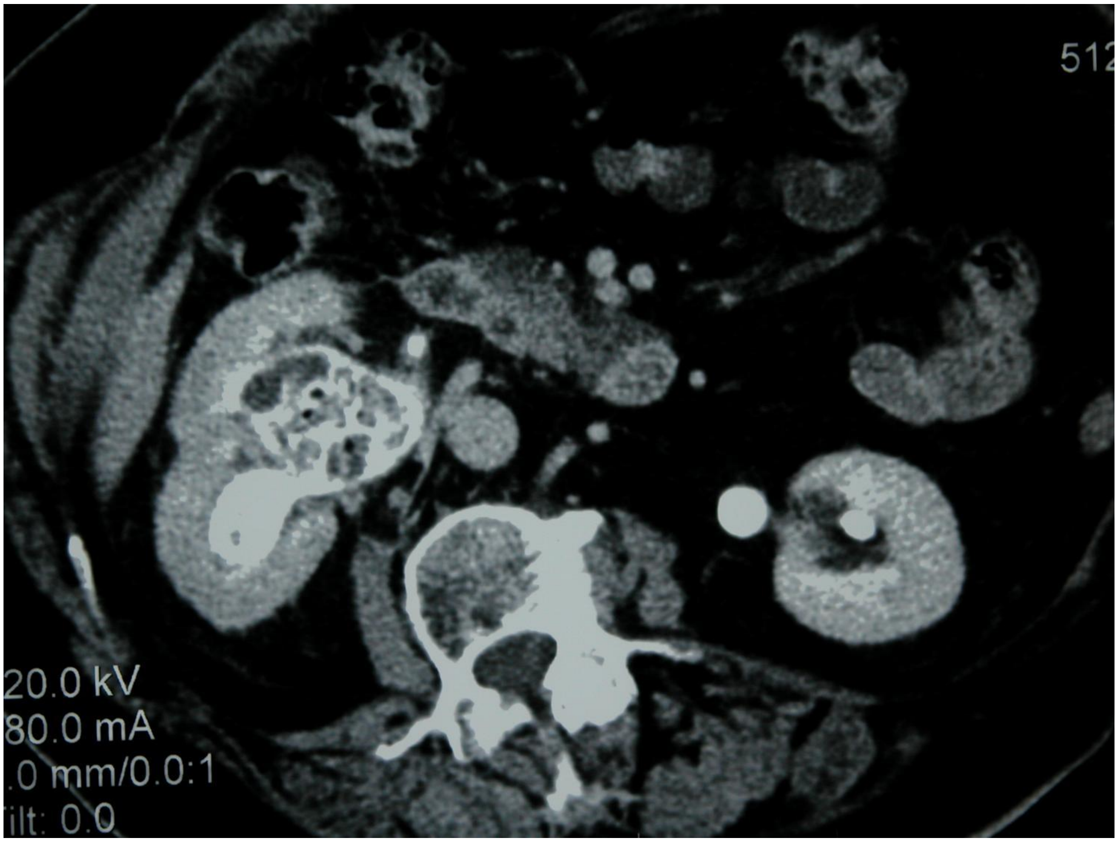

**FIGURE 1.** CT urography showing the extensive filling defect of the right renal pelvis and air bubbles.

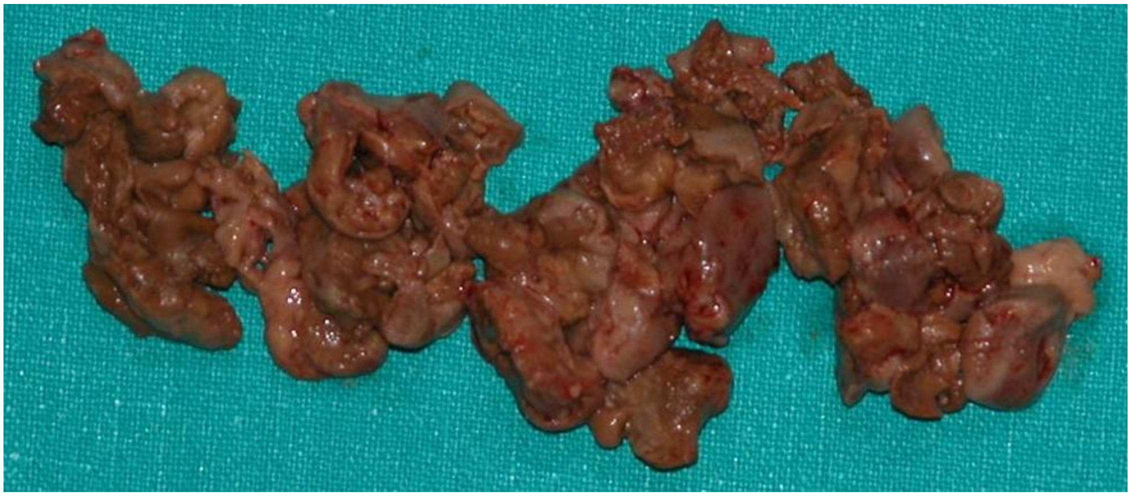

**FIGURE 2.** Amorphous and brown colored material: mycetoma by *C. albicans.*

## CASE 2

A 79-year-old woman, with previous history of urolithiasis, was referred to our department for right flank pain and fever. Ultrasound imaging revealed the presence of a hyperechoic and nonhomogeneous mass located in the renal right pelvis with concomitant hydronephrosis. Urinary cytology was negative. Intravenous urography revealed an extensive filling defect of a right renal enlarged pelvis due to the presence of a partially radiolucent mass (Fig. 3). CT urography showed that the pelvis was partially filled with water-like and positive-density material, mixed with small calcifications and gas bubbles (Fig. 4). Radiologic findings were highly suspicious for matrix urolithiasis. The patient underwent right open pyelotomy, which revealed the presence of brown and soft material mixed with blood clots and small calculi. Intraoperative appearance was highly suspicious for fungal infection and urine culture confirmed the presence of *C. albicans*. The postoperative course was uneventful and the patient was discharged on the 6th postoperative day. During hospitalization and, subsequently, at home (30 days postoperatively), the patient was administered antifungal therapy with fluconazole 400 mg daily. After 1 year, the patient is asymptomatic and urine culture is negative for fungal infection.

## DISCUSSION

Although mycotic infections of the urinary system occur not infrequently[1], obstruction of the upper urinary tract due to mycetomas or fungus balls is extremely rare[6]. Moreover, systemic infection and specific visceral involvement mainly occur in immunocompromised patients as a result of many causes. Interestingly, in the two cases reported, we did not find any predisposing condition possibly leading to a reduced immunologic competence and, to the best of our knowledge, these are the first cases reporting on fungus balls occurring in immunocompetent patients. Moreover, the presence of upper urinary tract obstruction, which is exceedingly rare, together with the immunologic integrity, led us to misdiagnosis due to the fact that both patients had a previous history of urolithiasis. Hence, in case of upper urinary tract obstruction with preoperative imaging features of the simultaneous presence of a low-density filling defect, calcified areas, and gas bubbles, the occurrence of fungus balls should be taken under consideration even in immunocompetent patients. Following urine culture confirmation, an adequate therapeutic strategy should be carried out, beginning with antifungal drugs, eventually followed by minimally invasive access to the pyelocaliceal system.

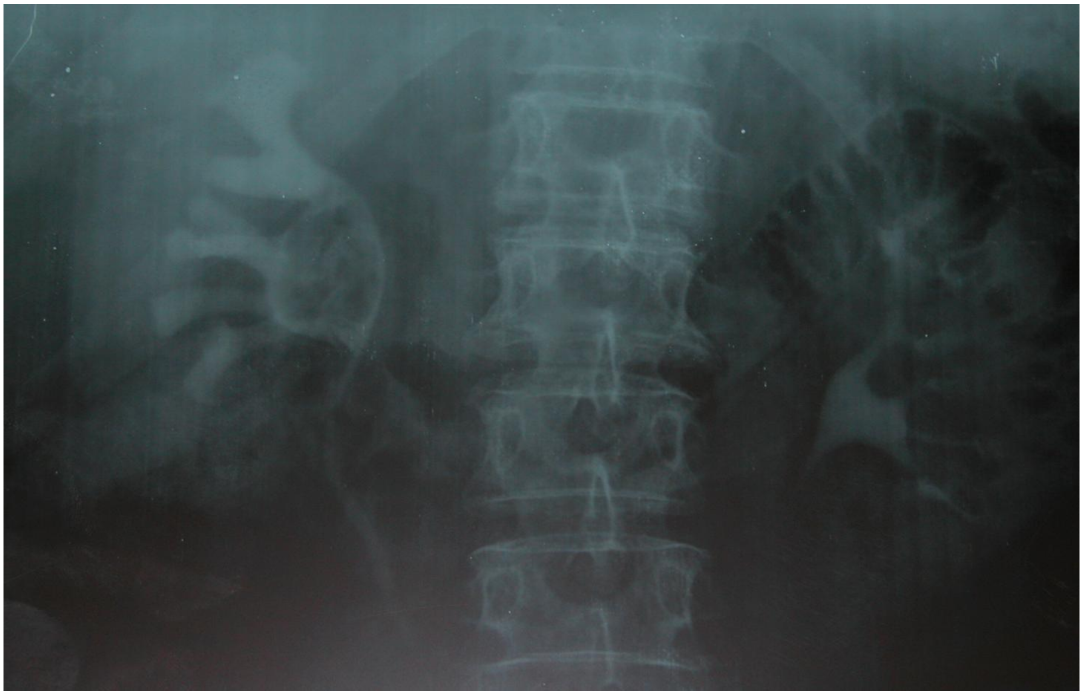

**FIGURE 3.** Intravenous urography: large filling defect of the renal pelvis due to partially radiolucent material.

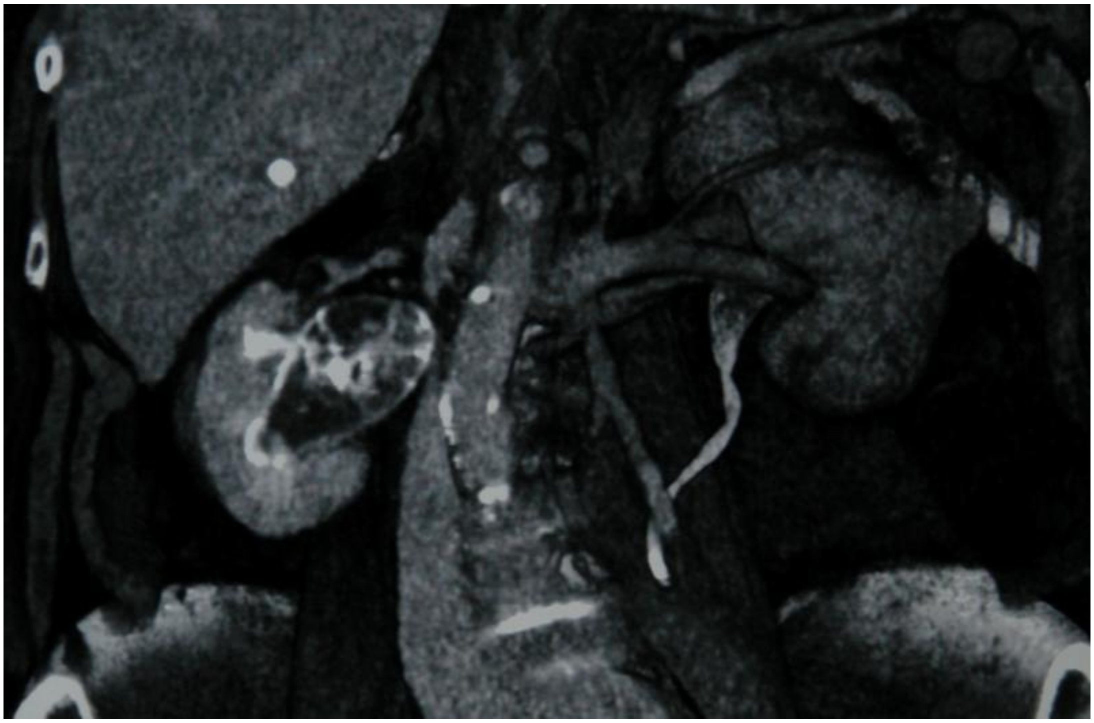

**FIGURE 4.** CT urography: the right renal pelvis is partially filled with water-like and positive-density material, mixed with small calcifications and air bubbles.
